# In Vitro Multiplication and NMR Fingerprinting of Rare *Veronica caucasica* M. Bieb

**DOI:** 10.3390/molecules26195888

**Published:** 2021-09-28

**Authors:** Desislava I. Mantovska, Miroslava K. Zhiponova, Milen I. Georgiev, Tsvetinka Grozdanova, Dessislava Gerginova, Kalina Alipieva, Svetlana Simova, Milena Popova, Veneta M. Kapchina-Toteva, Zhenya P. Yordanova

**Affiliations:** 1 Department of Plant Physiology, Faculty of Biology, Sofia University “St. Kliment Ohridski”, 8 Dragan Tsankov Blvd., 1164 Sofia, Bulgaria; d_mantovska@biofac.uni-sofia.bg (D.I.M.); zhiponova@biofac.uni-sofia.bg (M.K.Z.); veneta_kapchina@abv.bg (V.M.K.-T.); 2 Laboratory of Metabolomics, The Stephan Angeloff Institute of Microbiology, Bulgarian Academy of Sciences, 139 Ruski Blvd., 4000 Plovdiv, Bulgaria; milengeorgiev@gbg.bg; 3 Institute of Organic Chemistry with Centre of Phytochemistry, Bulgarian Academy of Sciences, bl. 9 Acad. Georgi Bonchev Str., 1113 Sofia, Bulgaria; tgrozdanova@orgchm.bas.bg (T.G.); dpg@orgchm.bas.bg (D.G.); alipieva@orgchm.bas.bg (K.A.); sds@orgchm.bas.bg (S.S.); popova@orgchm.bas.bg (M.P.)

**Keywords:** Plantaginaceae, in vitro cultivation, cytokinin, ROS, phenolic acids, iridoid glycosides

## Abstract

Micropropagation of rare *Veronica caucasica* M. Bieb. was achieved by successful in vitro cultivation of mono-nodal segments on MS medium supplemented with 1.0 mg L^–1^ 6-benzylaminopurine (BA) and then transferring the regenerated plants on hormone free basal MS medium for root development. In vitro multiplicated plants were successively acclimated in a growth chamber and a greenhouse with 92% survival. The number of plastid pigments and the total phenolics content in in vitro cultivated and ex vitro adapted plants were unchanged, and no accumulation of reactive oxygen species (ROS) was detected by staining with 3-3′-diaminobenzidine (DAB) and 2′,7′-dichlorofluorescein diacetate (DCF-DA). Nuclear Magnetic Resonance (NMR) fingerprinting allowed for the identification of the major alterations in metabolome of *V. caucasica* plants during the process of ex situ conservation. Iridoid glucosides such as verproside, aucubin and catalpol were characteristic for in vitro cultivated plants, while in ex vitro acclimated plants phenolic acid–protocatechuic acid and caffeic acid appeared dominant. The successful initiation of in vitro and ex vitro cultures is an alternative biotechnological approach for the preservation of *V. caucasica* and would allow for further studies of the biosynthetic potential of the species and the selection of lines with a high content of pharmaceutically valuable molecules and nutraceuticals.

## 1. Introduction

The genus *Veronica* (family Plantaginaceae) comprises more than 200 species distributed mainly in the Northern Hemisphere and the Australasian region, with centers of diversity in western Asia and New Zealand [1]. Most of the *Veronica* species are found in areas with a Mediterranean climate at different altitudes. In traditional medicine, leaves, flowers and roots of plants of the genus *Veronica* have been used to treat rheumatism [2], hemoptysis, laryngopharyngitis and hernia [3], besides respiratory diseases [4]. They also possess a wide range of biological activity such as antiscorbutic, diuretic, antimicrobial, antifungal, anti-inflammatory, scolicidal, anti-cancer, wound healing [5] and inhibitory potential on acetylcholinesterase, tyrosinase, lipoxygenase and xanthine oxidase [6]. Overall, based on these studies, *Veronica* species are a potential source of nutraceuticals and functional ingredients and hence good candidates for pharmacological applications.

Phytochemical studies of *Veronica* species revealed the presence of iridoid glucosides, phenylethanoid glycosides and acylated flavonoids, which are also considered as chemotaxonomic markers of the genus [7,8]. Arbutin, aucubin, catalpol [8,9] and catalpol derivatives such as veronicoside, verproside, amphicoside, verminoside, catalposide, 6-*O*-veratroylcatalposide, 6-*O*-isovanilloylcatalpol [10], mussaenoside and mussaenosidic acid esters [11,12] were identified as the main iridoid glucosides in representatives of the genus. Phenylethanoid glycosides such as verbascoside, isoverbascoside, lavandulifolioside and persicoside have been reported in *Veronica* [13]. Plenty of phenolic compounds were also identified, e.g., caffeic, ferulic, gallic, chlorogenic, protocatehuic and *p*-hydroxybenzoic acids and the flavonoids quercitrin, rutin, hyperoside, luteolin, myricetin, apigenin, chrysoeriol, hesperidin and its glucoside derivatives [9,12,14].

*Veronica caucasica* (IPNI, The International Plant Names Index) has limited distribution and is found only in the Northern Caucasus. Plant species with potential pharmacological properties are at high risk of being extinct in the near future. The Global Strategy for Plant Conservation (IUCN, 2002, updated 2011–2020) is the global authority for biodiversity conservation and aims to reduce and prevent the extinction of endangered species. The Strategy facilitates cooperation between the existing plant protection initiatives and supports the development of ex situ methods related to in situ conservation of rare and vulnerable species [15]. The micropropagation in in vitro conditions allows for the cultivation of plants in a controlled environment on nutrition medium in sterile conditions, regardless of seasons and climatic changes, and allows for the isolation of valuable secondary metabolites without disturbing the natural plant populations [16]. There are no available reports concerning ex situ conservation of *V. caucasica*, and the knowledge of its chemical composition is rather scarce. The aim of our study was to develop an efficient protocol for in vitro micropropagation of *V. caucasica* and subsequent adaptation in ex vitro conditions, as well as comparative NMR (Nuclear Magnetic Resonance) fingerprinting of in vitro and ex vitro cultivated plants.

## 2. Results

### 2.1. In Vitro Multiplication and Ex Vitro Acclimation of V. caucasica 

**Figure 1:**
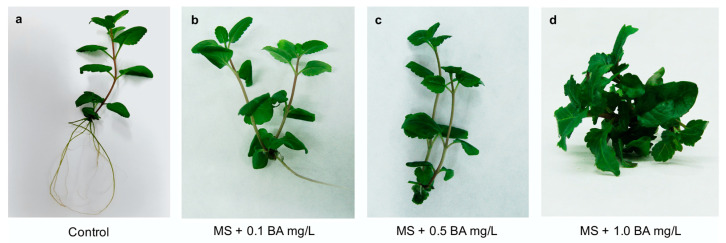
In vitro propagated *V. caucasica* plants. Control plant, in vitro cultivated on MS medium without growth regulators (**a**); in vitro cultivated plant on MS medium supplemented with 0.1 mg L^–1^ BA (**b**); in vitro cultivated plant on MS medium supplemented with 0.5 mg L^–1^ BA (**c**); in vitro cultivated plant on MS medium supplemented with 1.0 mg L^–1^ BA (**d**).

The *V. caucasica* shoot culture was induced by the sterilisation of 40 ripe dry seeds with 70% ethanol and subsequent washing with 96% ethanol. After 21 days, 35% of 20 seeds cultivated on ½ MS medium germinated, while these (20 seeds) inoculated on 0.7% water agar did not germinate. The sprouting seedlings were then transferred on basal MS medium, supplemented with 3% (*w*/*v*) sucrose and 0.7 g L^–1^ agar. After 25 days of cultivation, the regenerated *V. caucasica* plants showed a low growth index (1.5 ± 0.52 shoots per explant) but a well-developed root system (Table 1, Figure 1a).

**Figure 2:**
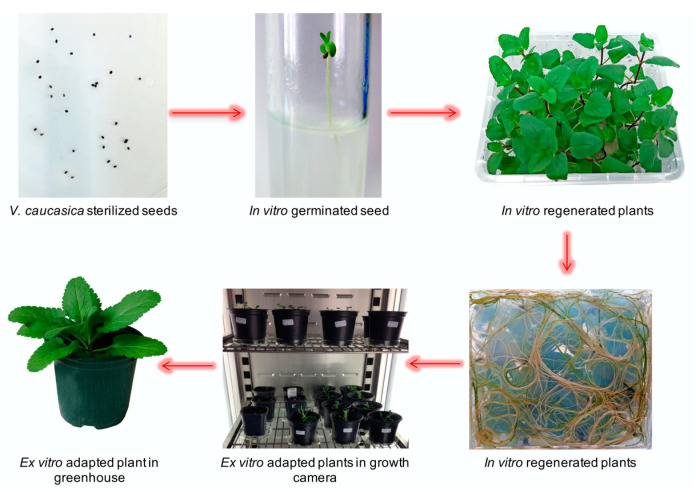
Ex situ conservation of *V. caucasica*.

In order to boost the plant growth, mono-nodal segments of regenerated *V. caucasica* plants were further inoculated on MS medium supplemented with increasing concentrations of the cytokinin BA (0.1 mg L^–1^, 0.5 mg L^–1^, 1 mg L^–1^). Within 20 days, media supplemented with 1.0 mg L^–1^ BA appeared more effective in promoting shoot development, hence ca. 96% of the explants showed shoot proliferation and produced 9.14 ± 0.64 shoots per explant (Table 1, Figure 1d). BA in concentration 0.1 and 0.5 mg L^–1^ did not significantly affect the shoot development but stimulated their elongation in ca. 93% of the explants (Table 1, Figure 1b,c). Higher concentrations of BA suppressed root formation and stimulated callus formation at the base of shoot tips. Further, micropropagated *V. caucasica* plantlets were transferred on hormone free MS medium for root development. Within 25 days, approximately 75% of regenerated micro-shoots managed to form thick and healthy roots with a maximum length of 5.20 ± 0.34 cm (Figure 2).

The in vitro grown plantlets that had 3 cm-long regenerated shoots and a well-developed root system were transferred into plastic pots containing a sterile soil. A one-month acclimation was maintained in a phytotron chamber under controlled environmental conditions and a gradual decrease in relative humidity. The survival rate of *V. caucasica* acclimated plants was relatively high—92%. Next, the ex vitro adapted plants were transferred to greenhouse and adapted for a period of six months (Figure 2). The percentage of surviving plants remained unchanged.

### 2.2. Comparative Determination of Plastid Pigments, Total Phenolics, Flavonoids and ROS Accumulation in In Vitro Cultivated and Ex Vitro Adapted Plants

**Figure 3:**
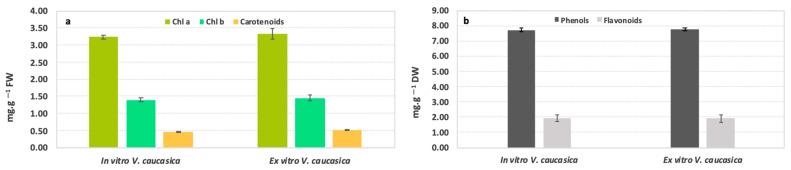
Comparative determination of some physiological indices in in vitro cultivated and ex vitro acclimated *V. caucasica* plants: plastid pigment content (**a**); total phenolic and flavonoid content (**b**). Data are presented as the mean ± SD.

To monitor the physiological status during the ex situ conservation of *V. caucasica* plants, leaves from in vitro and ex vitro adapted plants were analysed with respect to the content of plastid pigments (Figure 3). The overall ratio between Chl a and Chl b was 3 to 1, and comparison between the amounts of Chl a and Chl b showed no statistically significant difference between the examined variants (Figure 3a). The same tendency was noticed for the carotenoids content—their amounts were 6 and 3 times lower than Chl a and Chl b, respectively, and did not change between the in vitro and ex vitro variants: 0.47 ± 0.015 mg g^–1^ FW and 0.52 ± 0.020 mg g^–1^ FW, respectively (Figure 3a).

**Figure 4:**
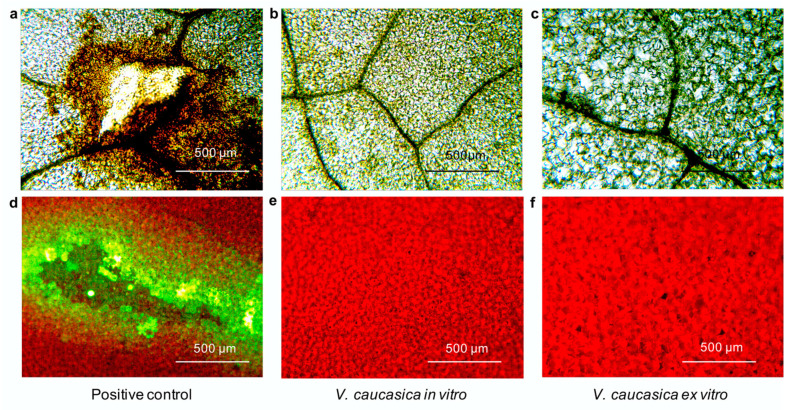
Hydrogen peroxide and ROS accumulation in leaves of in vitro cultivated and ex vitro adapted *V. caucasica* plants: DAB staining—H_2_O_2_ is stained in brown, intensity of the colour corresponds to H_2_O_2_ amount (**a**–**c**). Epifluorescence of ROS after staining with DCF-DA. The green fluorescence indicates presence of ROS; Chl fluorescence appears in red (**d**–**f**). Positive controls, mechanically injured (**a**,**d**). Scale bar = 500 µm.

In *V. caucasica*, no statistically significant changes in the number of total phenolics and flavonoids were observed in in vitro cultivated (7.73 ± 0.10 and 1.91 ± 0.21 mg g^–1^ DW, respectively) and ex vitro adapted (7.75 ± 0.09 and 1.92 ± 0.25 mg g^–1^ DW, respectively) plants (Figure 3b). Next, the accumulation of ROS in leaves from the studied *V. caucasica* variants was checked by DAB staining and DCF-DA fluorescent dye (Figure 4). Intensive DAB staining and massive green fluorescence of the marker dye, which indicate the presence of H_2_O_2_ and ROS, were observed only in the positive controls subjected to mechanical injury (Figure 4a,d). No signal was noticed in intact leaves of in vitro cultivated and ex vitro adapted plants, hence referring to a normal physiological state (Figure 4b,c,e,f).

### 2.3. NMR-Based Metabolite Profiling of In Vitro Cultivated and Ex Vitro Acclimated V. caucasica 

**Figure 5:**
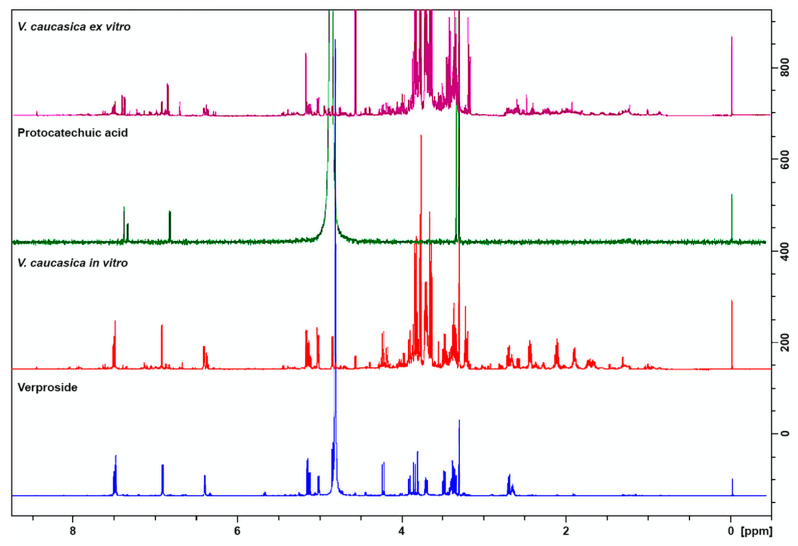
^1^H NMR spectra of in vitro cultivated and ex vitro adapted *V. caucasica* plants, pure verproside and protocatechuic acid.

In order to reveal the metabolic alterations in *V. caucasica* plants during the process of ex situ conservation, NMR analyses were performed. Both qualitative and quantitative differences were established between in vitro cultivated and ex vitro acclimated plants (Figure 5, Table 2).

A variety of primary and secondary metabolites such as amino- and organic acids, carbohydrates, phenolic compounds and iridoid glucosides were identified in the samples (Table 2). Signals of iridoid glucosides, amino acids (valine, leucine, isoleucine) and malic acid appeared characteristic for in vitro cultivated plants, while ex vitro acclimated plants were abundant in phenolic compounds (Table 2). The iridoid glucoside verproside was the main secondary metabolite identified in in vitro cultivated plants. The presence of catalpol and aucubin was confirmed accordingly. In ex vitro acclimated *V. caucasica* plants, the protocatechuic acid (3,4-dihydroxybenzoic acid) was dominant, while caffeic acid was detected in both samples in relatively equal amounts. The identification of verproside, protocatechuic acid, catalpol and aucubin was supported by comparison with authentic samples.

## 3. Discussion

### 3.1. In Vitro Multiplication and Ex Vitro Acclimation of V. caucasica 

The high importance of bioactive molecules produced by *Veronica* sp. and the limited distribution of *V. caucasica* focused our efforts on developing alternative approaches for plant growth and sustainable mass production of valuable secondary metabolites. The micropropagation is an essential method for ex situ conservation of rare and threatened plant species, species with reduced populations and low fertility, as well as for the fast propagation of valuable medicinal plants without ecological impact on their natural populations [16,17]. In this study, we managed to initiate *V. caucasica* in vitro culture from seeds. Due to the low growth index (number of shoots per explant) we added series of concentrations of the BA phytohormone to the basal medium and established 1 mg L^−1^ BA (i.e., 4 µM) as optimal for shoot development. The effective response to BA in shoot regeneration was reported earlier for *V. anagallis-aquatica*, where 0.5 µM BA stimulated the formation of 43.7 ± 1.85 shoots per explant after six weeks of cultivation in 85% of the nodal explants [18]. Higher concentration of BA (above 0.5 µM) dramatically inhibited shoot proliferation and shoot length. The effect of BA in promoting shoot multiplication has been established in various medicinal plants such as *Cardiospermum halicacabum* [19], *Lavandula viridis* [20], *Eclipta alba* [21], *Verbascum eriophorum* [22], *Achillea thracica* [16], *Artemisia annua* [23], and *Stachys leucoglossa* [17]. The regenerated *V. caucasica* plantlets formed thick and healthy roots up to 5.20 ± 0.34 cm in length on hormone free MS medium. In contrast, micropropagated *V. anagallis-aquatica* shoots failed to induce rooting on MS medium without plant growth factors, and successful root formation has been achieved on MS medium supplemented with 0.5 µM naphthaleneacetic acid (NAA) with a maximum root length of 2.8 ± 0.14 cm [18]. The well-developed root system is a prerequisite for successful ex vitro adaptation [23], and up to 92% was reached for *V. caucasica*.

### 3.2. Physiological State of V. caucasica Plants during Ex Situ Conservation

It has been demonstrated that during in vitro cultivation the plant growth is slowed down because of the reduced rate of photosynthesis due to slower electron transport in the thylakoid membranes and lower CO_2_ assimilation [24]. The physiological status during the ex situ conservation of *V. caucasica* plants was assessed in leaves from in vitro and ex vitro adapted plants based on the total content of plastid pigments and secondary metabolites such as phenolics and flavonoids and by screening the ROS accumulation. The results showed no statistically significant difference between the examined variants. The 3 to 1 ratio between Chl *a* and Chl *b* was evidence for a good physiological state of both *V. caucasica* variants [25]. The observed consistency in the pigment content is an indication of good regeneration potential of the species during the processes of ex situ conservation. Previous report pointed the pigment content as a good marker to discriminate between low-temperature tolerant and sensitive *Hypericum* sp. grown in vitro—a decrease in chlorophylls was observed in the stress-sensitive population [26]. On the other hand, the increased carotenoid content was suggested to have a protective role against oxidative stress by preventing membranes damage from ROS [25]. Dragolova et al. [27] also observed similar pigment content between *Nepeta nuda* plants grown in in vitro and ex vitro conditions, which was further correlated with successful regeneration.

Phenolic compounds are among the largest group within the plant metabolites pool. Along with the wide range of biological activities, phenolics have been recognised as major antioxidant agents owing to their structural characteristics and chemical behavior. Based on their hydrogen-donating ability, phenolic compounds may act as free-radical scavengers and, consequently, exert a protective effect against ROS [28,29]. During in vitro cultivation of *Lamium album* and *A. thracica*, the metabolic potential was strongly inhibited; however, it recovered upon ex vitro adaptation [16,24]. In other plant model systems, the number of total phenolics and flavonoids was reported to decrease significantly in in vitro cultivated plants compared to those adapted ex vitro or in vivo grown [30,31,32]. The lack of significant changes between the *V. caucasica* variants suggests that the phytochemical potential eventually remains constant during the stages of ex situ conservation of this medicinal plant species. In low-temperature sensitive *Hypericum* sp. grown in vitro, the stress did not affect the phenolics quantity; however, it caused elevated lipid peroxidation and respective accumulation of H_2_O_2_ in the tissues [26]. In plants subjected to biotic and abiotic stress, in planta accumulation of ROS has been detected by DAB staining and DCF-DA fluorescent dye [33,34]. In support of the normal physiological state in the *V. caucasica* variants, both approaches indicated no signal in intact leaves.

### 3.3. Metabolite Profiling during V. caucasica Ex Situ Conservation

The metabolite profiling of *V. caucasica* plants during the process of ex situ conservation demonstrated sensitivity with respect to the primary and secondary metabolite composition. The amino acids (valine, leucine, isoleucine), the malic acid and the iridoid glucosides were characteristic for in vitro cultivated plants, and in ex vitro acclimated plants, the phenolic compounds prevailed. The accumulation of the branched-chain amino acids valine, leucine and isoleucine has been related to enhanced cell division activity and growth [35]. The higher level of primary metabolites could be explained with more active biosynthetic processes during in vitro cultivation, which needs further validation. In addition, the iridoid glucosides verproside, catalpol and aucubin, which are characteristic molecules of *Veronica* sp., were identified in in vitro cultivated plants. The compound verproside is a catalpol derivative with anti-inflammatory, antioxidant and antinociceptive activities [36] and is a potent anti-asthmatic/COPD drug candidate, acting by blocking the TNF-α/NF-κB signaling pathway [37,38]. Verproside has been also reported in *V. cuneifolia* subsp. *cuneifolia* and *V. cymbalaria* [10], *V. (Hebe) brachysiphon*, *V. (Hebe) odora* [8], *V. austriaca*, *V. (Hebe) ligustrifolia*, *Veronica (Hebe) salicifolia* and *Veronica (Hebe) andersonii* [7]. Aucubin has been also found in *V. cuneifolia* subsp. *cuneifolia* and *V. cymbalaria* [10], *V. (Hebe) brachysiphon*, *V. (Hebe) odora* [8] and *V. persica* [13]. In ex vitro, acclimated *V. caucasica* plants predominated the protocatechuic acid (3,4-dihydroxybenzoic acid), which was identified as a main compound in the phenolics pool in *V. montana*, showed effective antibacterial activity against six pathogenic bacteria and was successfully used to preserve cream cheese by inhibiting the growth of *Listeria monocytogenes* [39]. In ex vitro conditions, the plants are exposed to more severe environmental changes that require the synthesis of phenolic compounds to aid the plant adaptation [40].

## 4. Materials and Methods

### 4.1. Plant Material and In Vitro Cultivation of V. caucasica 

*Veronica caucasica* M. Bieb. seeds were obtained from the Medicinal Plant Garden, Department of Pharmacognosy with Medicinal Plant Unit (Medical University of Lublin, Poland). The plant material originates from Botanical Garden, Leipzig University, Germany (accession number 886). In vitro shoot culture of *V. caucasica* was induced by sterilising 40 ripe dry seeds with 70% ethanol for 5 min, followed by washing with 96% ethanol for 10 s. Under aseptic conditions, the sterilised seeds were inoculated in equal parts on half-strength MS medium [41] and on 0.7% water agar. After 21 days of germination, the seedlings were transferred on basal MS medium supplemented with 3% sucrose and 0.7% agar and in vitro cultivated under controlled environmental conditions (16 h light/8 h dark, 60 µmol m^–2^ s^–1^ photosynthetic photon flux density, Philips TLD-33, temperature 25 °C and 60–70% relative air humidity). Shoot proliferation was induced by the transferring of mono-nodal segments from regenerated plants on MS medium supplemented with increasing concentrations of the cytokinin 6-benzylaminopurine (BA)–0.1 mg L^–1^, 0.5 mg L^–1^ and 1.0 mg L^–1^ (i.e., 0.4 µM; 2 µM and 4 µM, respectively). After 20 days, regenerated plants were transferred on hormone-free basal MS medium for rooting and micropropagated twice under controlled conditions for 25 days each. Fully expanded leaves from the 2nd or 3rd nodes of the shoot were collected from in vitro regenerated plants and used for further biochemical studies and NMR-based metabolite profiling. 

### 4.2. Ex Vitro Acclimation 

In vitro cultivated plants that had 3–4 cm long regenerated shoots with 2–3 internodes and a well-developed root system were subjected to ex vitro adaptation. Regenerated plants were transferred into plastic pots containing a mixture from sterile soil substrate (peat:coconut fibers:sand = 2:1:1). A one-month acclimation was maintained in a phytotron chamber (POL-EKO APARATURA SP.J.A. Polok–Kowalska KK 350 STD 1400 W) with 16/8 h light/dark, 100 µmol m^–2^ s^–1^ PPFD, 22 ± 2 °C, the relative humidity was decreased from 90% to 60% every week. After 30 days of acclimation, the adapted plants were transferred to greenhouse while maintaining a temperature in the range of 18–26 °C, humidity of the environment 55–65% and a photoperiod in accordance with the seasonal variability. After six months of acclimation, newly formed fully expanded leaves from the 2nd or 3rd nodes of the stem of ex vitro plants were harvested and used for further biochemical studies and NMR-metabolic profiling.

### 4.3. Plastid Pigments Quantification

Samples of 50 mg fresh leaf material were homogenised with 80% acetone and centrifuged at 10,000 g for 10 min. Further, the pigment extracts were subjected to a spectrophotometric quantification analysis of chlorophyll (Chl) Chl *a* (at 663 nm), Chl *b* (at 645 nm) and carotenoids (at 452.5 nm) with a Shimadzu 1800 UV spectrophotometer. The calculation of the concentrations of Chl *a*, Chl *b* and carotenoids was performed according to the equations of McKinney [42].

### 4.4. ROS Imaging

The H_2_O_2_ accumulation in the leaf was visualised by 3,3’-diaminobenzidine (DAB) according to [43] with modifications. Leaf segments were excised and placed in 50 mL tubes covered with 0.1% (*w/v*) DAB and incubated for 18 h on an orbital shaker. The dye was then poured off and the chlorophyll was removed by boiling the samples in 96% ethanol. DAB is rapidly absorbed by plant tissues and polymerises in the presence of H_2_O_2_, giving a visible brown color with an intensity corresponding to the amount of H_2_O_2_. The samples were observed under a Nikon Eclipse microscope, TS 100 and images were taken with a Nikon DXM 1200 digital camera. 

In addition to DAB staining, intracellular ROS were detected using 2’7’-dichlorofluorescein diacetate (DCF-DA), according to [44] with modifications. To determine intracellular ROS, leaf segments from in vitro cultivated and ex vitro acclimated plants were isolated and incubated on an orbital shaker for 60 min in the presence of 10 µm DCF-DA. The fluorescence was determined by a Nikon Eclipse microscope, TS 100, filter B-2A, exciter 450–490, DM 505, BA 520, equipped with a Nikon DXM 1200 digital camera (Nikon Inc., Melville, NY, USA).

### 4.5. Determination of Total Phenolics Content 

Total phenolics content was determined according to [45] with some slight modifications. Fifty milligrams of dried leaf material were homogenised with 5 mL methanol followed by incubation in an ultrasonic bath for 5 min and centrifuged at 9000 rpm for 15 min. The reaction mixture contains 0.1 mL plant extract, 1.5 mL Folin Ciocalteu’s Reagent (diluted 1:10 with dH_2_O) and 1.4 mL Na_2_CO_3_. The samples were incubated in dark for 30 min at room temperature. The absorbance was measured at λ = 756 nm with spectrophotometer Shimadzu 1800 UV. The total phenolic quantity was quantified by standard curve using gallic acid as a standard, and it is expressed as gallic acid equivalents mg per gram of dry weight (mg GAE.g^–1^ DW).

### 4.6. Determination of Flavonoids Content

The total flavonoids content was determined according to [46] with modifications. Briefly, 50 mg dried leaf material were homogenised with 5 mL methanol followed by incubation in an ultrasonic bath for 5 min and centrifuged at 9000 rpm for 15 min. The reaction mixture contains 0.25 mL plant extract, 0.75 mL methanol, 0.05 mL 10% AlCl_3,_ 0.05 mL 1M CH_3_COOK and 1.4 mL dH_2_O. The samples were vortexed and incubated at room temperature for 30 min. Absorbance was measured at λ = 415 nm with a spectrophotometer Shimadzu 1800 UV. The flavonoid content was quantified by standard curve using quercetin as a standard, and it is expressed as quercetin equivalents per mg per gram of dry weight (mg QE g^–1^ DW).

### 4.7. Extraction Procedure and NMR Analyses

Samples of fresh leaf material were air dried and 50 mg of each 5 biological replicates were homogenised with equal amounts of CD_3_OD (0.75 mL) and D_2_O (0.75 mL KH_2_PO_4_ buffer, pH 6.0), containing 0.005% (*w*/*v*) trimethylsilyl propanoic acid (TSPA-*d_4_*). After 20 min ultrasonication (35 kHz; UCI-50Raypa^®^ R. Espinar S.L., Barcelona, Spain) samples were centrifuged (14,000× *g*, 20 min). Then, the supernatants were transferred to 5 mm glass walled NMR tubes and analysed at the NMR spectrometer [47,48]. Briefly, proton (^1^H) as well as 2D NMR spectra (*J*-resolved, COSY, HSQC) were recorded at 25 °C on an AVII+ 600 spectrometer (Bruker, Karlsruhe, Germany), operating at a proton NMR frequency of 600.01 MHz [47]. Deuterated methanol was used for internal lock. The resulting ^1^H NMR spectra for each sample were further processed by referencing to the internal standard TSPA, phased and baseline corrected, by running TopSpin software (3.6.5, Bruker BioSpin Group). CD_3_OD and D_2_O from Deutero GmbH (Kastellaun, Germany) were used in the experiments.

### 4.8. Data Analysis 

The presented data are mean values with standard deviation (SD) of at least 12 scores (3 repetitions per variant in each of 4 independent sets of experiments). The graphic figures were generated using MS Excel Version 16.16.7. Data were processed by one-way analysis of variance (ANOVA) followed by Holm-Sidak’s statistical test at a probability level of *p* ≤ 0.05, which was performed by Sigma Plot 11.0 software.

## 5. Conclusions

The herein developed protocol for in vitro multiplication of *V. caucasica* comprises the germination of sterilised ripe dry seeds on ½ MS medium and the cultivation of sprouting seedlings on hormone free MS medium for 25 days. The optimal induction of shoot proliferation from mono-nodal segments was achieved on MS medium supplemented with 1.0 mg L^–1^ BA for a period of 20 days, and the subsequent transferring of regenerated plants on hormone free MS medium for root development and accumulation of leaf biomass for 25 days. The in vitro cultivated and ex vitro adapted plants did not differ in the content of plastid pigments and total phenolic compounds, and the markers for oxidative stress did not fluctuate. NMR fingerprinting allowed for the identification of major metabolic alterations in *V. caucasica* plants during the process of ex situ conservation. Verproside, a promising molecule with valuable pharmacological activities for human health, appeared the most abundant metabolite from the iridoid glucosides pool of in vitro cultivated plants, while in ex vitro adapted plants, protocatechuic acid was dominant. The established collections from in vitro tissue and ex vitro cultures of *V. caucasica* could be used for future studies of the biosynthetic potential of the species and the application of biotechnological approaches to increase the yield of pharmaceutically valuable molecules and nutraceuticals.

## Figures and Tables

**Table 1:** Influence of BA on length and number of shoots, root formation and callogenesis of in vitro propagated *V. caucasica*.

Variants	Length of Shoots (cm)	Number of Shoots	Root Formation	Length of Roots (cm)	Degree of Callus Formation
Control	2.85 ^a^ ± 0.35	1.5 ^a^ ± 0.52	+	4.97 ^a^ ± 0.85	–
BA 0.1 mg L^–1^	4.80 ^b^ ± 0.85	2.0 ^ab^ ± 0.07	+	2.36 ^b^ ± 1.24	+
BA 0.5 mg L^–1^	4.32 ^b^ ± 1.22	2.5 ^b^ ± 0.71	–	–	++
BA 1.0 mg L^–1^	0.90 ^c^ ± 0.40	9.14 ^c^ ± 0.64	–	–	++

+ Weak callus formation (below 0.5 cm in diameter at the base of shoot). ++ Significant callus formation (over 0.5 cm in diameter at the base of shoot). Values represent mean ± SD of three repeated experiments with 10 replicates each. One-way ANOVA (Holm-Sidak) statistical test is applied to estimate the difference between all the variants as indicated by letters. Different letters ^a,b,c^ indicate significant difference between the variants (*p* ≤ 0.05).

**Table 2:** Chemical shifts (*δ*) and coupling constants (*J*) of the metabolites identified in *V. caucasica* plants by their relevant 1D and 2D NMR spectra.

Metabolite	*V.c.* In Vitro ^a^	*V.c.* Ex Vitro ^a^	Selected Signals, Multiplicity and Coupling Constant ^b^
***Amino acids***			
Alanine	++	+	*δ* 1.47 (d, *J* = 7.2)
Valine	+	-	*δ* 0.99 (d, *J* = 7.0)/*δ* 1.04 (d, *J* = 7.0)
Leucine	+	-	*δ* 0.95 (d, *J* = 6.3)/*δ* 0.97 (d, *J* = 6.3)
Isoleucine	+	-	*δ* 0.94 (t, *J* = 7.3)/*δ* 1.01 (d, *J* = 7.3)
***Sugars***			
α-Glucose	+	++	*δ* 5.17 (d, *J =* 3.8)
β-Glucose	+	++	*δ* 4.56 (d, *J =* 7.9)/3.18 (dd, *J =* 7.9, 9.2)
Sucrose	+	+	*δ* 5.39 (d, *J =* 3.9)
***Organic acids***			
Acetic acid	-	+	*δ* 1.93 (s)
Lactic acid	++	+	*δ* 1.31 (d, *J* = 6.9)/*δ* 4.06 m
Succinic acid	-	+	*δ* 2.48 (s)
Formic acid	+	+	*δ* 8.45 (s)
Malic acid	+	-	*δ* 2.80 (dd, *J* = 16.9, 8.2)/*δ* 2.93 (dd, J = 16.9, 3.9)
***Phenolic acids***			
Caffeic acid	++	++	*δ* 7.63 (d, *J* = 16.0)/*δ* 7.14 (d, *J* = 2.2)/*δ* 7.05 (dd, *J* = 8.5, 2.2)/*δ* 6.88 (d, *J* = 8.4)/*δ* 6.28 (d, *J* = 16.0)
Protocatechuic acid	++	++++	*δ* 7.41 (d, *J* = 2.1)/*δ* 7.38 (dd, *J* = 8.4,2.1)/*δ* 6.85 (d, *J* = 8.3)
***Iridoid glucosides***			
Verproside	+++	+	*δ* 7.51 (dd, *J* = 8.3, 2.2)/*δ* 7.48 (d, *J* = 2.2)/*δ* 6.92 (d, *J* = 8.3)/*δ* 6.41 (dd, *J* = 6.1, 2.0)/*δ* 5.16 (d, *J* = 9.7)/*δ* 5.13 (dd, *J* = 6.0, 1.7)/*δ* 5.02 (d, *J* = 10.0)/*δ* 4.85 (d, *J* = 7.9)/*δ* 4.23 (d, *J* = 13.3)/*δ* 2.70 (m)/*δ* 2.66 (m)
Aucubin	++	+	*δ* 6.32 (dd, *J* = 6.2, 1.9)/*δ* 5.81 (brs)/*δ* 5.11 (dd, *J* = 6.2, 3.7)/*δ* 5.09 (d, *J* = 6.1)/*δ* 4.34 (d, *J =* 15.6)/*δ* 4.22 (d, *J* = 15.2)
Catalpol	++	++	*δ* 6.38 (dd, *J* = 6.0, 1.7)/*δ* 5.11 (dd, *J* = 6.0, 4.6)/*δ* 5.03 (d, *J* = 9.9)/*δ* 4.18 (d, *J =* 13.3)/*δ* 3.98 (dd, 8.0, 1.1)/*δ* 3.78 (d, 13.3) /*δ* 2.58 (dd, *J* = 9.6, 7.7)/*δ* 2.27 m
***Others***			
Choline	+	+	*δ* 3.19 (s)

*V.c.* in vitro—*V. caucasica* in vitro; *V.c.* ex vitro—*V. caucasica* ex vitro. ^a^ The number of “+” refers to relative fold differences and “–” to absence of the particular compound. ^b^ Proton NMR chemical shifts (*δ*) and coupling constant (*J*).

## Data Availability

The data presented in this study are available on request from the corresponding author.

## Sample Availability

Samples of the compounds are available from the authors.
